# Effects of L-lysine aescinat on intracranial pressure in patients with severe traumatic brain injury

**DOI:** 10.1186/2197-425X-3-S1-A853

**Published:** 2015-10-01

**Authors:** VV Krylov, SS Petrikov, A Solodov, SA Badygov, ED Mekhia Mekhia

**Affiliations:** Sklifosovsky Research Institute, Neurosurgery, Moscow, Russian Federation; Sklifosovsky Research Institute, Neuro ICU, Moscow, Russian Federation; Sklifosovsky Research Institute, ICU, Moscow, Russian Federation

## Introduction

Increased intracranial pressure (ICP) results in cerebral blood flow decrease and cerebral edema formation. Correction of intracranial hypertension (ICH) is one of the most important goals of intensive care in patients with severe traumatic brain injury (TBI).

## Objectives

To determine the effects of L-lysine aescinat on ICP in patients with severe TBI.

## Methods

Twenty patients with TBI and Glasgow coma scale below 9 enrolled in the study. Mean age was 39.3 ± 13.6 years, male/female - 18/2. All patients were operated: 6 patients underwent craniotomy and intracranial hematoma removing; 11 - decompressive craniotomy and intracranial hematoma removing. In 3 patients only ICP-sensor was implanted. ICP-monitoring was used in all patients. Ten patients were randomized to L-lysine aescinat treatment (daily dose of 20 ml for 7 days after surgery) (study group), 10 - to standard therapy (control group). We perfomed a comparative analysis of the mean ICP and the incidence of ICH within 7 days after surgery in the study and control groups.

## Results

The length of ICP monitoring was 6.4 ± 3.7 days: in the control group - 7.6 ± 4.9 days, in the study group - 5.2 ± 1.4 days. Mean intracranial pressure (mmHg) was less in the study group as compared to patients in the control group: 1^st^ day - 14.3 ± 5.9 (n = 136) vs 17.0 ± 3.8 (n = 156), 2^nd^ day - 15.7 ± 5.4 (n = 158) vs 17.4 ± 4.6 (n = 141), 3^rd^ day - 17.0 ± 6.5 (n = 144) vs 17.1 ± 6.9 (n = 161), 4^th^ day - 17.3 ± 9.5 (n = 163) vs 20.7 ± 5.9 (n = 135), 5^th^ day - 14.6 ± 5.9 (n = 83) vs 18.9 ± 5.6 (n = 136), 6^th^ day - 12.8 ± 4.5 (n = 79) vs 15.9 ± 6.4 (n = 66), 7^th^ day - 12.4 ± 8.6 (n = 56) vs 14.4 ± 7.6 (n = 95). The number of intracranial hypertension episodes was higher in the control group compared with patients who received L-lysine aescinat: 1^st^ day - 4.2 ± 4.2 vs 2.8 ± 3.3, 2^nd^ day - 6.5 ± 5.1 vs 3.0 ± 2.5, 3^rd^ day - 6.0 ± 8.0 vs 2.6 ± 3.3, 4^th^ day - 9.6 ± 10.1 vs 3.4 ± 3.8, 5^th^ day - 7.4 ± 8.2 vs 2.6 ± 3.7, 6^th^ day - 7.3 ± 8.6 vs 0.4 ± 0.9, 7^th^ day - 5.0 ± 9.3 vs 1.3 ± 1.3.

## Conclusions

L-lysine aescinat treatment in patients with severe traumatic brain injury is accompanied by reduction of mean intracranial pressure and the number of intracranial hypertension episodes.

